# Ondansetron-induced QT prolongation among various age groups: a systematic review and meta-analysis

**DOI:** 10.1186/s43044-023-00385-y

**Published:** 2023-07-03

**Authors:** Kamaldeep Singh, Arpit Jain, Ishita Panchal, Hritik Madan, Anubhav Gupta, Aakanksha Sharma, Surabhi Gupta, Anastas Kostojchin, Anmol Singh, Ishanjit Singh Sandhu, Jayesh Mittal, Loveleen Bhogal, Shiny Teja Kolli, Vishal Reddy Bejugam, Salil Chaturvedi, Akhil Bhalla, Shobhit Piplani

**Affiliations:** 1 Department of Medicine, KAHER’s Jawaharlal Nehru Medical College, Belagavi, Karnataka 590 010 India; 2 Department of Medicine, Adesh Medical College and Hospital, Ambala, 136 135 India; 3 Department of Internal Medicine, Icahn School of Medicine, Mount Sinai West Hospital, New York, NY 10019 USA; 4 Department of Internal Medicine, Christian Medical College, Ludhiana, Punjab 141008 India; 5 Department of Medicine, Icahn School of Medicine at Mount Sinai/James J. Peters VA Medical Center, Bronx, NY 10468 USA; 6 Department of Cardiology, Government Medical College and Hospital, Sector 32, Chandigarh, 160030 India; 7 Emergency Medicine, Canadian Red Cross, Ottawa, ON K2P 2H8 Canada; 8 Department of Internal Medicine, St Mary Mercy Hospital Livonia, Trinity Health, Livonia, MI 48154 USA; 9 Department of Medicine, Medstar Washington Hospital Center, Washington, DC 20010 USA; 10 Department of Medicine, Jacobi Medical Center/North Central Bronx, Albert Einstein College of Medicine, NYC Health and Hospitals, Bronx, NY 10467 USA; 11 Department of Anesthesiology, Adesh Medical College and Hospital, Ambala, 136 135 India

**Keywords:** QT prolongation, Ondansetron, Arrhythmia, Drug safety

## Abstract

**Background:**

Ondansetron is a selective 5-hydroxytryptamine type 3 serotonin-receptor antagonist with antiemetic properties used inadvertently in the emergency department for controlling nausea. However, ondansetron is linked with a number of adverse effects, including prolongation of the QT interval. Therefore, the purpose of this meta-analysis was to assess the occurrence of QT prolongation in pediatric, adult, and elderly patients receiving oral or intravenously administered ondansetron.

**Methods:**

A thorough electronic search was conducted on PubMed (Medline) and Cochrane Library from the databases' inception to August 10, 2022. Only those studies were considered in which ondansetron was administered orally or intravenously to participants for the treatment of nausea and vomiting. The prevalence of QT prolongation in multiple predefined age groups was the outcome variable. Analyses were conducted using Review manager 5.4 (Cochrane collaboration, 2020).

**Results:**

A total of 10 studies involving 687 ondansetron group participants were statistically analyzed. The administration of ondansetron was associated with a statistically significant prevalence of QT prolongation in all age groups. An age-wise subgroup analysis was conducted which revealed that the prevalence of QT prolongation among participants younger than 18 years was not statistically significant, whereas it was statistically significant among participants aged 18–50 years and among patients older than 50 years.

**Conclusions:**

The present meta-analysis provides further evidence that oral or intravenous administration of Ondansetron may lead to QT prolongation, particularly among patients older than 18 years of age.

## Background

Ondansetron has antiemetic effects as a selective 5-hydroxytryptamine type 3 (5-HT3) serotonin-receptor antagonist [1]. Between 2013 and 2019, the number of Ondansetron prescriptions in the USA increased twofold, from 6,516,077 prescriptions in 2013 to 11,856,066 prescriptions in 2019, while the number of patients increased marginally [2].

It is one of the most common and widely prescribed medications for the prevention and treatment of postoperative and chemotherapy-induced nausea and vomiting. Moreover, it has demonstrated efficacy in the treatment of acute gastroenteritis in children brought to the emergency department and irritable bowel syndrome with diarrhea [3–5]. During a 3-year study period, it was estimated that Ondansetron was administered to 19,857 (58.2%) patients on their initial visit to the pediatric emergency department, and a prescription was written for 11,624 (34.1%) patients [6]. In addition, the proportion of patients receiving ondansetron has increased over time (11.8% in 2006, 62.5% in 2015), both in the emergency department (10.6% in 2006, 55.5% in 2015) and as outpatient prescriptions (3.3% in 2006, 45.3% in 2015) with little or no change in hospitalizations over the same time period [7]. Pediatricians are increasingly prescribing Ondansetron in the emergency department for conditions other than acute gastroenteritis, including fever, appendicitis, and respiratory pathologies [8].

Furthermore, Ondansetron is commonly used as an antiemetic during pregnancy, particularly during the first trimester, despite inconclusive evidence of its safety for the mother and the child [9, 10].

The most frequently occurring side effects include migraine-type headache, dry mouth, malaise, and constipation [1, 11]. Furthermore, ondansetron is linked to pruritus, tiredness, and increased liver function tests, all of which are established consequences of liver disease [12]. Ondansetron, a 5-hydroxytryptamine-3 receptor antagonist, has also shown pro and anticonvulsant effects in animal studies [13].

Orthostatic hypotension, lengthening of the QT interval (associated with Torsades de pointes), and other electrocardigram segments and various arrhythmias are the most common cardiovascular side effects linked with ondansetron [14]. In September 2011, the Food and Drug Administration (FDA) published a message warning that ondansetron administration at doses higher than those frequently used in the emergency department may cause lethal arrhythmia [15]. The following year, this warning was revised to include the risk of QT prolongation associated with intravenous administration of 32 mg of ondansetron [16]. This risk, however, has not been uniformly reported among various age groups and there have been mixed results on whether QT prolongation is a significant side-effect of ondansetron.

The purpose of this meta-analysis was to assess the occurrence of QT prolongation in pediatric, adult, and elderly patients receiving oral or intravenous doses of ondansetron under the currently defined safe dose of 32 mg.

## Methods

### Search strategy and data sources

This single-arm meta-analysis was completed in close adherence with the Preferred Reporting Items for Systematic Reviews and Meta-Analyses (PRISMA) guidelines statement [17].

A comprehensive electronic search was performed on PubMed (Medline) and Cochrane Library from the inception of these databases till August 10, 2022. An extensive search strategy was formulated by the combination of the Medical Subject Headings (MESH terms) “Long QT syndrome” OR “LQTS” OR “QT prolongation” OR “Torsades de pointes” AND “Ondansetron” OR “Zofran” OR “Emeset” OR “5-HT3 receptor antagonist” OR “serotonin receptor antagonists” OR “serotonin blockers.”

### Eligibility criteria

The inclusion criteria are based on PICOS: P (Patients): Any patients treated with ondansetron; I (Intervention): ondansetron; C (Control): None; O (Outcome): prevalence of QT prolongation in multiple predefined age groups; S (Studies): Randomized Controlled Trials and Observational studies, published in English.

Only those articles were considered for inclusion in our review that excluded patients from their studies who were receiving any other drug or treatment option that had the potential to cause QT prolongation.

We did not include any of the articles that were originally published in languages other than English. In addition, all different kinds of reviews, case reports, case series, cross-sectional studies, editorials, commentaries, and studies based on animals were not included in this research in any capacity. Each article was individually assessed by the authors to assure eligibility according to the inclusion criteria. Missing data were excluded from consideration in the study.

### Data extraction

Data were extracted by the first author and reviewed independently by other authors. Articles yielded from the electronic search were exported to EndNote Reference Library software in order to eliminate duplicates. The relevance of studies was initially determined based on the title and abstract, and then the full text was examined. The following baseline characteristics were extracted: the name of the first author, the year of publication, the type of study, the number of participants and their mean age, the dosage of the intervention, the percentage of males in the ondansetron group, and the QT at baseline. On an Excel spreadsheet, baseline attributes were extracted. Table 1 contains the characteristics of the baseline. The primary outcome was a mean difference in QT interval after administration of Ondansetron.

**Table 1:** Study characteristics of included studies

Author’s name, year	Type of study	Number of participants	Average age	Males (%)	Dosage of ondansetron and Route of administration	Baseline QT	
Charbit et al. [20]	Observational study	42	44 ± 16 years	40.5	4 mg Intravenous	439 + 29 ms	
Grecu et al. [21]	Randomized double-blind trial	135	51.9 ± 13.9 years	29.6	4 mg Intravenous	N/A	
Rosow et al. [22]	Randomized trial	125	54 ± 15 years	33.6	4 mg Intravenous	N/A	
Ganjare et al. [23]	Prospective, randomized, single-blind study	37	47.14 ± 9.2 years	N/A	8 mg Intravenous	N/A	
Zuo et al. [24]	Randomized trial	58	29 years (range 18–45 years)	37.9	8 mg Intravenous	409.6 + 16.72 ms	
Jamwal et al. [25]	Randomized trial	58	38.32 ± 10.59 years	0	4 mg Intravenous	N/A	
Hoffman et al. [26]	Retrospective study	134	47.8 months (4.8–168 months)	46	0.15 mg/kg Intravenous	415 ms; 95% CI 343–565 ms	
Campleman et al. [27]	Prospective multicenter cohort study	8	36 years (26–50 years)	N/A	N/A	N/A	
Yang et al. [28]	Retrospective observational study	80	53.3 months (7–161 months)	45	0.18 mg/kg Oral	403.3 ± 24.0 ms	
Sutherland et al. [29]	Prospective study	52	> 18 years	47	4 mg Intravenous	439 +/− 38 ms	

### Quality assessment

The revised Cochrane Risk of Bias (RoB 2) tool was used to examine the quality of the included Randomized controlled trials (RCTs) [18]. Reports were analyzed for generation of random sequence, randomization of participants to exposure, blinding of participants personnel and outcome assessors, selective reporting of outcomes, and missing data.

The Newcastle–Ottawa Scale was used to assess the quality of observational studies [19]. This scale uses a “star” system to assess the quality of a study in two domains: selection of participants and ascertainment of outcomes of interest. Cohort studies were evaluated out of a total score of 8. A study is considered of good quality if there are 3 or 4 stars in the selection domain AND 1 or 2 stars in the comparability domain AND 2 or 3 stars in the outcome/exposure domain. A study is considered of fair quality if there are 2 stars in the selection domain AND 1 or 2 stars in the comparability domain AND 2 or 3 stars in the outcome/exposure domain. A study is considered of poor quality if there are 0 or 1 stars in the selection domain OR 0 stars in the comparability domain OR 0 or 1 stars in the outcome/exposure domain. No study was excluded based on quality alone.

### Statistical analyses

**Fig. 1:**
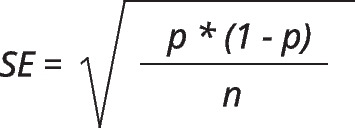
Formula used for standard error calculation

Analyses were done using the Review manager 5.4 (Cochrane collaboration, 2020) tool. Prevalence was calculated through raw data. This along with other extracted information was used to find standard errors using the formula in Fig. 1.

Where “*p*” was the prevalence and “*n*” was the number of people aware of screening or people not screened. The prevalence and standard error from each study were then input into Review Manager through the inverse variance method to compute pooled prevalence along with a 95% confidence interval.

Heterogeneity was measured using the Higgins *I*^2^ statistics and was reported as a percentage for every outcome. For an *I*^2^ value of less than 50%, low heterogeneity was indicated, moderate heterogeneity was considered when the *I*^2^ value was less than 75%, and high heterogeneity was observed with an *I*^2^ value of greater than 75%. Outcomes reporting an *I*^2^ greater than 75% were subjected to sensitivity analysis to determine the individual effects of each study on a certain pooled outcome.

## Results

### Systemic search

**Fig. 2:**
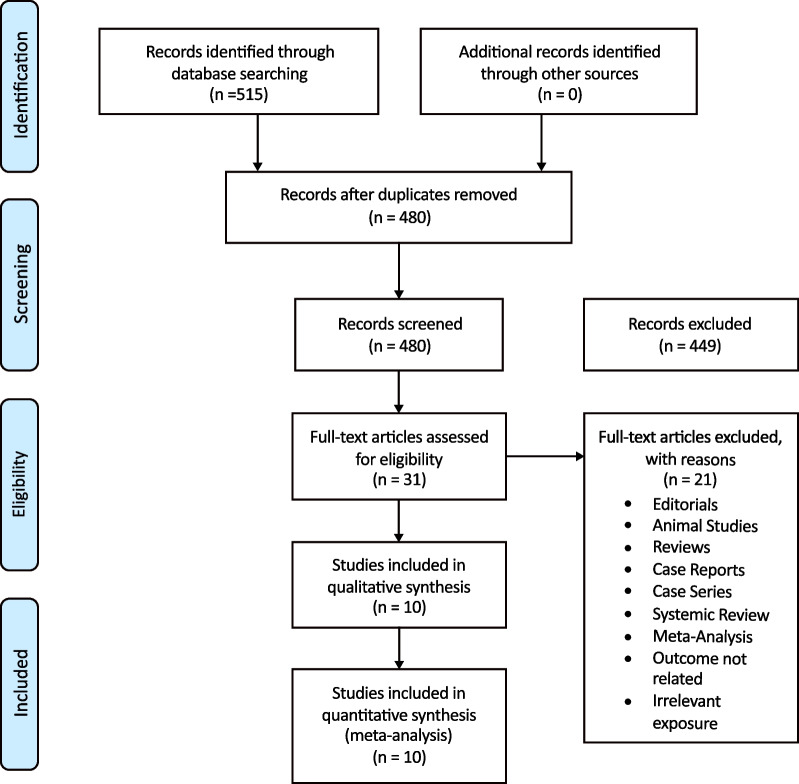
Prisma flowchart

A comprehensive search on 2 databases yielded a total of 515 results. Considering the study eligibility criteria, 10 studies were selected for the meta-analysis, as shown in Fig. 2.

### Study characteristics

A total of ten studies met the inclusion criteria and out of these five were randomized controlled trials and the rest were cohort studies [20–29]. These studies selected for the statistical analysis consisted of 687 participants in the ondansetron group. The mean ages of the patients in the pooled sample ranged between 4 and 75+ years. Study characteristics of the included studies are given in Table 1. Considering the prevalence of QT prolongation as the primary outcome, baseline values of QT were also included in Table 1.

### Meta-analysis results

**Fig. 3:**
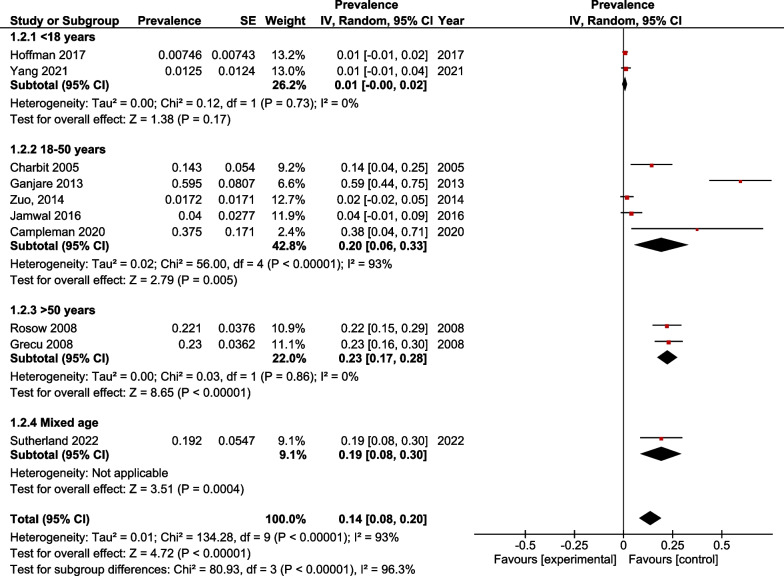
Prevalence of QT prolongation

The results of our meta-analysis are illustrated in Fig. 3. A statistically significant prevalence of QT prolongation was associated with the administration of ondansetron in all age groups (Prevalence, 0.14; 95% CI, 0.08 to 0.20; *p* value < 0.00001; *I*^2^ = 96.3%). A high overall in-study heterogeneity was reported.

### Subgroup analysis

A subgroup analysis was performed to assess the prevalence of QT prolongation associated with ondansetron administration in different age groups. Two studies were included in the < 18 years subgroup [26, 28]. A statistically non-significant prevalence of QT prolongation among participants younger than 18 years was reported (Prevalence, 0.01; 95% CI, − 0.00 to 0.02; *p* value = 0.17; *I*^2^ = 0%) and no in-study heterogeneity was observed. A total of five studies included participants in the 18–50 years age group [20, 23–25, 27]. The incidence was QT prolongation among participants of this age group was statistically significant (Prevalence, 0.20; 95% CI, 0.06 to 0.33; *p* value = 0.005; *I*^2^ = 93%) and a high study heterogeneity was reported. Two studies were included in the > 50 years subgroup [21, 22]. A statistically significant prevalence of QT prolongation among participants older than 50 years was reported (Prevalence, 0.23; 95% CI, 0.17 to 0.28; *p* value < 0.00001; *I*^2^ = 0%) and no in-study heterogeneity was observed. The prevalence of QT prolongation among participants of the study included in the mixed age group was statistically significant (Prevalence, 0.19; 95% CI, 0.08 to 0.30; *p* value = 0.00004).

### Leave-one-out sensitivity analysis

**Fig. 4:**
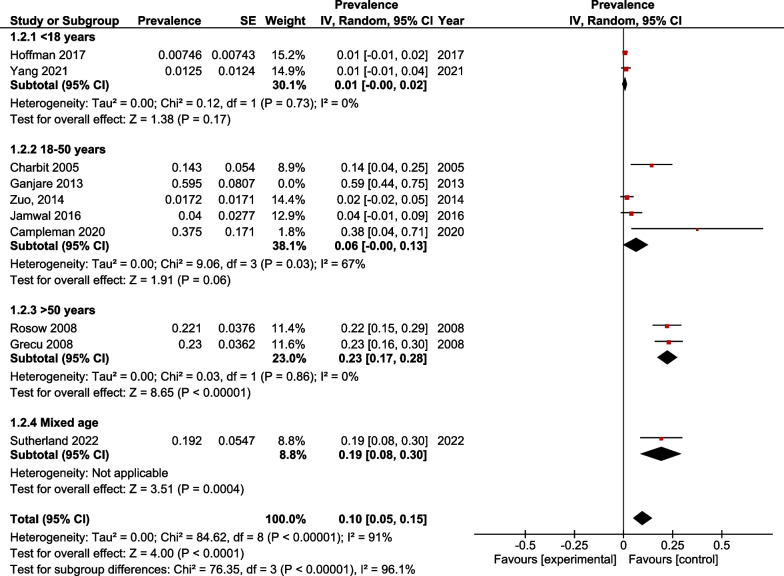
Forest plot for leave-one-out sensitivity analysis showing a change of significance in 18–50 year age subgroup

Excluding the studies one-by-one from the 18–50 years subgroup did not reduce the in-study heterogeneity (*I*^2^ = 67% *p* value = 0.06). However, the prevalence of QT prolongation among participants among the 18–50 years subgroup was statistically non-significant upon the exclusion of the study conducted by Ganjare et al. [23] but at a lower confidence interval limit of 0.00. The results of the sensitivity analysis are depicted in Fig. 4.

### Publication bias

**Fig. 5:**
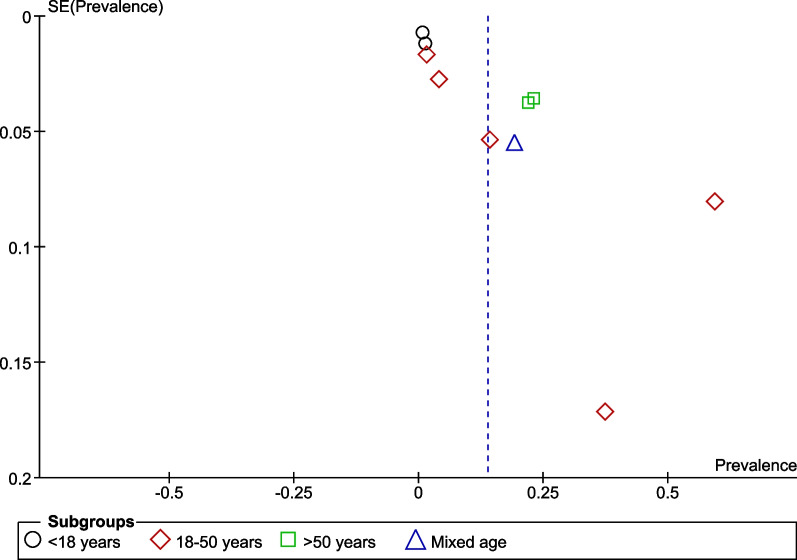
Funnel plot

On visual assessment, the funnel plot was asymmetrical suggesting publication bias among the included studies, as demonstrated in Fig. 5.

### Quality assessment

**Fig. 6:**
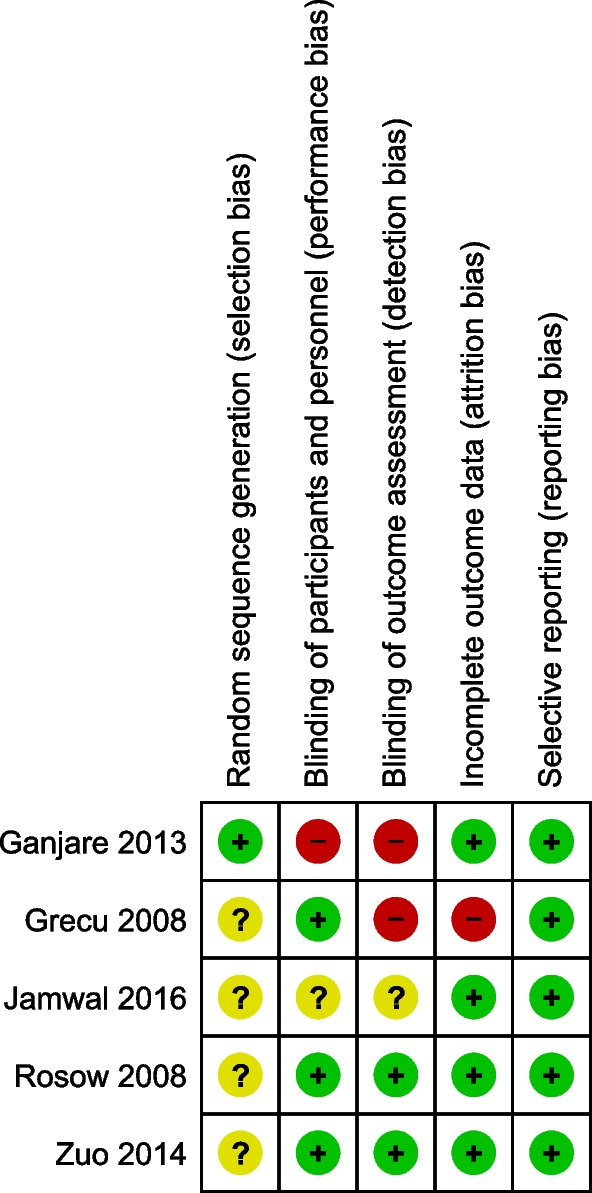
Quality assessment of RCTs using revised Cochrane risk-of-bias tool

Quality assessment of RCTs and cohort studies is presented in Fig. 6 and Table 2, respectively. 4 out of the 5 cohort studies were of poor quality and only one study was of fair quality.

**Table 2:** Quality assessment of cohorts using New Ottawa Scale

	Charbit et al	Hoffman et al	Campleman et al	Yang et al	Sutherland et al
Selection	*	**	**	**	*
Comparability					
Outcome	***	**			

### Limitations

The small number of participants along with limited follow-up, included in this meta-analysis, limited the reliability of the results. In addition, the dosage and route of ondansetron administration were not standardized across the included studies, compromising the validity of the findings. Furthermore, a statistical increase in QT may or may not be reflected clinically, thus determination of clinical outcome needs to be performed across varying dosages.

## Discussion

Ondansetron is an antiemetic used to prevent and treat nausea and vomiting that may be caused by anesthesia during surgery, chemotherapy in cancer patients, and pregnancy. In addition, ondansetron is increasingly utilized in the emergency department, particularly for pediatric patients with acute gastroenteritis. Oral administration of ondansetron to such patients in the emergency department not only reduces vomiting but also decreases intravenous fluid administration and hospitalization frequency. In addition to acute gastroenteritis, it has been observed that pediatricians frequently prescribe ondansetron for conditions such as fever, appendicitis, and respiratory pathologies.

However, ondansetron is associated with numerous adverse effects, including headache, fatigue, pruritus, teratogenic risk in pregnant women, and QT prolongation. QT interval measures the time between ventricular depolarization and repolarization on an electrocardiogram [30]. This corresponds to the beginning of the QRS complex through the end of the T wave [31]. Some factors that either lengthen or shorten the QT interval include adrenergic stimuli, heart rate, and drugs [23]. QT prolongation is a serious condition that is frequently associated with an increased risk of atrial fibrillation and Torsades de Pointes (TdP), a polymorphic ventricular tachycardia that can lead to fatal ventricular fibrillation [31]. It has been observed that the normal range of the QT interval varies with heart rate, increasing during bradycardia and decreasing during tachycardia, for example. Thus, QT interval must be 'corrected' for accurate results, particularly in hospital settings where patients' resting heart rates may not be normal [31]. The QT interval after correction is known as QT. In this meta-analysis, the correlation between ondansetron administration and the incidence of QT prolongation in the following age groups was investigated for the following age groups: less than 18 years, 18 to 50 years, older than 50 years, and mixed age group.

In studies by Grecu et al. [21] and Rosow et al. [22] patients with cardiac arrhythmias, seizure disorders, Parkinson's disease, those receiving treatment with dopamine antagonists, and dexamethasone were excluded [21, 22].

In studies by Ganjare et al. [23], Hoffman et al. [26], Yang et al. [28] and Sutherland et al. [29] patients with baseline prolonged QT interval, arrhythmias, and serum electrolyte imbalance were excluded. Furthermore, in study [25], patients who had received prior antiemetics before surgery, administration of steroids, psychotropic drugs, pregnant or lactating women, those with prolonged QT interval, bundle branch block, patients undergoing chemotherapy, and those allergic to the study drugs were excluded [23, 26, 28, 29].

Additionally, in the study conducted by Yang et al. [28], patients with oliguria, surgical abdomen, congenital heart disease, arrhythmias, or a history of prolonged use of QT prolongation medication were excluded [28].

Even though the results of this meta-analysis demonstrate a significant correlation between the incidence of QT prolongation and the administration of ondansetron, the high heterogeneity between studies undermines the validity of these findings. As demonstrated by the results of the sensitivity analysis, this heterogeneity was not reduced by excluding studies individually, further minimizing the confidence in the findings. The inclusion of poor-quality studies can introduce bias and compromise the overall validity of the conclusions drawn in the meta-analysis.

Considering the limited data available and the inclusion of studies with poor quality, it is essential to acknowledge the potential limitations of the research paper. These limitations could affect the generalizability and reliability of the conclusions. Future research should aim to address these limitations by including a larger number of high-quality studies that provide comprehensive data on the baseline QT interval. This would strengthen the validity and reliability of the findings in the field of ondansetron-induced QT prolongation across various age groups.

In addition, subgroup analysis revealed a statistically significant correlation between ondansetron administration and the incidence of QT prolongation in patients aged 18 to 50 years, over 50 years, and mixed age groups. There was no in-study heterogeneity among the subgroup of participants older than 50 years, enhancing the validity of the significant findings. In contrast, the sensitivity analysis did not reduce the high in-study heterogeneity among the '18 to 50-year-old' subgroup. In addition, the exclusion of the Ganjare et al. study rendered the results of this subgroup non-significant (*p* value = 0.06) [23].

The two studies by Grecu et al. [21] and Rosow et al. [22], which included the old age patients, suggest that the most probable explanation for higher incidence of ondansterone induced QT prolongation are:

1. Age-related changes: As individuals age, there are natural changes that occur in the cardiovascular system. These changes include alterations in the structure and function of the heart, such as fibrosis, decreased compliance, and changes in ion channels. These age-related changes can affect the electrical conduction system of the heart, making older individuals more susceptible to drug-induced QT prolongation. The altered electrical properties of the aging heart can amplify the effects of medications like ondansetron on the QT interval.
2. Polypharmacy: Older adults often take multiple medications to manage various health conditions, a phenomenon known as polypharmacy. The simultaneous use of multiple drugs increases the risk of drug interactions. Some medications, when combined with ondansetron, can further increase the risk of QT prolongation. Certain medications, such as other antiemetics, antipsychotics, antibiotics, and certain antidepressants, can interfere with the same cardiac ion channels affected by ondansetron, compounding the risk of QT prolongation.
3. Underlying medical conditions: older adults are more likely to have pre-existing medical conditions, such as heart disease, hypertension, electrolyte imbalances, or liver and kidney dysfunction. These conditions can impair the body's ability to process medications, including ondansetron, and increase the risk of QT prolongation. For example, electrolyte imbalances, particularly low levels of potassium, magnesium, or calcium, can disrupt the electrical activity of the heart and contribute to QT prolongation.
4. Reduced physiological reserve: older adults generally have reduced physiological reserves and diminished organ function compared to younger individuals. This reduced reserve can make older adults less capable of compensating for any adverse effects caused by medications like ondansetron. Age-related declines in organ function, particularly in the liver and kidneys, can impair drug metabolism and elimination, leading to higher drug concentrations and prolonged drug effects, including QT interval prolongation.

The exact mechanism by which ondansetron mediates the prolongation of the QT interval is still unknown. However, it has been hypothesized that 5-Hydrotryptamine-3 (5HT3) receptor antagonists block cardiac sodium channels, widening the QT interval. This hypothesis was investigated in the study conducted by Klooster et al. [32]. Effects of 5-Hydrotryptamine-3 (5HT3) receptor antagonists on human α-subunit Nav1.5 (cardiac sodium channel) heterologously expressed in HEK293 cells were assessed, and it was demonstrated that all 5-Hydrotryptamine-3 (5HT3) receptor antagonists including ondansetron inhibit Nav1.5 in a concentration and state-dependent manner [32].

The findings of our study are in contrast to the results of a network meta-analysis that included a total of 97,516 randomized participants and 44 single drugs. No or minimal effect of ondansetron administration on QT prolongation was demonstrated. Moreover, the reliability of these results was enhanced by the large sample size and no in-study heterogeneity (*I*^2^ = 0%) further compromising the authenticity of the results of our meta-analysis [33]. In another systematic review and network meta-analysis, the safety and efficacy of 5-Hydrotryptamine-3 (5HT3) receptor antagonists were assessed. Contrary to the findings of our study, no significant correlation between ondansetron administration and QT prolongation was drawn in this meta [34]. However, the credibility of this study was hindered by the limited number of included studies that reported QT prolongation and the small sample size of the included studies [34].

Interesting findings were reported by Tricco et al. in their study comparing the safety and efficacy of multiple 5-Hydrotryptamine-3 (5HT3) receptor antagonists in patients undergoing chemotherapy. The risk of QT prolongation was significantly greater in the dolasetron + dexamethasone group as compared to the ondansetron + dexamethasone group [35]. It has been postulated that co-administration of dexamethasone could positively influence the impact of ondansetron on QT interval and hence, these results are not comparable with the findings of our study.

This meta-analysis included two studies that evaluated the incidence of QT prolongation in pediatric patients with acute gastroenteritis [26, 28]. In both of these studies, QT prolongation was observed in only one patient, indicating that there is no correlation between ondansetron administration and QT prolongation in the pediatric population. This finding, along with the absence of within-study heterogeneity, enhanced the reliability of the statistically non-significant (*p* = 0.17) subgroup analysis results from this meta-analysis. In contrast, oral administration was used in the study conducted by Yang et al. [28], whereas intravenous administration was used in the study conducted by Hoffman et al. [26]. Variability in the mode of administration could undermine the validity of these results. In addition, an observational study of 100 pediatric patients failed to find any correlation between the administration of ondansetron and the incidence of QT prolongation [36].

The administration of ondansetron has been linked to QT prolongation, a potentially serious cardiac condition. However, the validity of this association is uncertain due to high heterogeneity among studies. Although certain medications have the capability to extend the duration of the QT interval, which is a measurement indicating the heart's electrical activity, there is no guarantee that they will lead to dangerous disruptions in heart rhythm. Numerous elements contribute to the emergence of irregular heartbeats, including the individual's underlying health status and the existence of other risk factors. Moreover, the likelihood of encountering serious heart rhythms differs among various medications, with certain medications carrying a greater probability than others. In clinical practice, healthcare professionals should carefully consider the potential risk of QT prolongation associated with ondansetron administration, particularly in patients within the identified age groups. Patient-specific factors, such as underlying cardiac conditions, concurrent medications, and individual risk profiles, should be taken into account when assessing the overall benefit-risk balance of using ondansetron as an antiemetic. Close monitoring of cardiac parameters and prompt intervention in case of any signs or symptoms of QT prolongation are crucial.

## Conclusions

This meta-analysis provides evidence that oral or intravenous administration of ondansetron, an antiemetic, may cause QT prolongation, especially in patients older than 18 years. There was no statistically significant effect of ondansetron in children younger than 18 years old, although caution must be exercised because cases have been reported in this age group as well. Therefore, we advise using ondansetron with caution, particularly in patients with a history of heart disease. To thoroughly validate our present findings, additional large-scale, multicenter studies evaluating additional parameters are required.

## Data Availability

Not applicable.

## Abbreviations

CI: Confidence Interval
5HT3: 5-Hydroxytryptamine-3
FDA: Food and Drug Administration
RCT: Randomized Controlled Trial
